# Long non-coding RNA GAS5 accelerates oxidative stress in melanoma cells by rescuing EZH2-mediated CDKN1C downregulation

**DOI:** 10.1186/s12935-020-01167-1

**Published:** 2020-04-09

**Authors:** Wei Xu, Zeqiang Yan, Fen Hu, Wei Wei, Chao Yang, Zhihua Sun

**Affiliations:** 1 Department of Dermatology, Xiangyang Central Hospital, Affiliated Hospital of Hubei University of Arts and Science, Xiangyang, 441021 People’s Republic of China; 2 Department of Gastroenterology, Xiangyang Central Hospital, Affiliated Hospital of Hubei University of Arts and Science, Xiangyang, 441021 People’s Republic of China; 3 Department of Oncology, Xiangyang Central Hospital, Affiliated Hospital of Hubei University of Arts and Science, Dongjin District, Xiangyang, 441021 People’s Republic of China

**Keywords:** Long non-coding RNA GAS5, EZH2, H3K27me3, CDKN1C, Melanoma, Oxidative stress

## Abstract

**Background:**

The significance of long non-coding RNAs (lncRNAs) in mediating oxidative stress of cancers has been implicated recently. This study proposed a potential therapeutic target lncRNA growth arrest-specific transcript 5 (GAS5) for melanoma, due to its crucial role in oxidative stress and apoptosis of melanoma cells by regulating the enhancer of zeste homolog 2 (EZH2)-mediated CDKN1C expression.

**Methods:**

The lncRNA GAS5 expression pattern was examined in melanoma tissues and cells. The correlation of lncRNA GAS5, EZH2, and CDKN1C with survival rate of melanoma patients was analyzed. In melanoma cell lines, lncRNA GAS5 expression was overexpressed or knocked down to clarify its effects on cell viability, apoptosis, and oxidative stress. The interaction between lncRNA GAS5 and EZH2 was examined by RIP and RNA pull-down assays followed by verification of the target relationship between EZH2 and CDKN1C.

**Results:**

High expression of EZH2 and poor expression of lncRNA GAS5 and CDKN1C was observed in melanoma tissues and found to be correlated with the reduction in survival expectancy of melanoma patients. Overexpression of lncRNA GAS5 or CDKN1C or EZH2 knockdown could inhibit cell viability but enhance melanoma cell apoptosis and oxidative stress. Importantly, lncRNA GAS5 attenuated EZH2 expression by recruiting E2F4 to the EZH2 promoter region and knockdown of EZH2 upregulated CDKN1C expression by inhibiting the H3K27me3.

**Conclusion:**

The evidence provided by our study highlighted the involvement of lncRNA GAS5 in the translational suppression of EZH2 as well as the upregulation of CDKN1C, resulting in the promotion of melanoma cell apoptosis and oxidative stress.

## Background

The majority of melanomas are generally regarded as a kind of deadly skin-related malignancy that severely affects the eyes and gastrointestinal mucous and other parts of the body [1]. Melanoma originates from melanocytes (responsible for pigment formation in the basal layer of the epidermis) [2]. Characterized by aggressive proliferation, invasion and early metastasis [3], melanoma remains resistant to both chemotherapy and radiotherapy [4]. The fact that the incidence of malignant melanoma tends to be increasing in the last few decades however, in most patients, if diagnosed at the early stage, is likely to be cured [5]. Though surgery remains the main and definitive treatment for early-stage melanoma but it’s rarely curative for the advanced stages of melanomas [6]. Importantly, oxidative stress has been reported to be associated with the transformation and progression of melanoma [7] and the induction of oxidative stress could inhibit distant metastasis in circulating melanoma cells in vivo [8]. Additionally, long noncoding RNAs (lncRNAs) have been confirmed to exert a critical role in the regulation of tumorigenesis and the progression of melanoma [9].

LncRNA growth arrest specific transcript 5 (GAS5) has been revealed to play a vital role in progression of non-small-cell lung cancer [10]. LncRNA GAS5 has been indicated to function as a tumor-inhibitor lncRNA, due to its role in stimulating growth arrest as well as apoptosis [11]. Furthermore, lncRNA GAS5 has been observed to be remarkably diminished in gastric cancer tissues, while the ectopic expression of lncRNA GAS5 was verified to suppress gastric cancer cell proliferation and accelerate apoptosis in vitro and in vivo [12]. Importantly, lncRNA GAS5 was unraveled to act as a tumor suppressor in the progression of melanoma [13]. Moreover, lncRNA GAS5 was reported to suppress enhancer of zeste homolog 2 (EZH2) transcription via recruiting E2F4 to EZH2 promoter to induce bladder cancer cell apoptosis [14]. EZH2, the critical constituent of the Polycomb repressive complex 2 (PRC2), suppresses gene expression and modulates cell proliferation and differentiation during embryonic development [15]. A previously reported study has revealed that EZH2 exhibits high expression in melanoma and is associated with its development [16]. Interestingly, in the present study, the dual-luciferase reporter gene assay, revealed that EZH2 could negatively regulate the expression of clin-dependent kinase inhibitor 1C (CDKN1C). CDKN1C (also known as p57^KIP2^), one of the CDK inhibitors of the Cip/Kip family, has been associated with various cellular processes of cancer [17]. The silencing of CDKN1C has been shown to contribute to the progression of colorectal carcinoma [18]. Based on this above discussion, we hypothesized that lncRNA GAS5 acts as a suppressor in melanoma and its underlying mechanism could involve EZH2 and CDKN1C. Thus, this study was designed to further testify this hypothesis and to investigate the underlying regulatory molecular mechanism of lncRNA GAS5 in melanoma cell oxidative stress concerning EZH2 and CDKN1C, to present a theoretical foundation for an enhanced understanding of melanoma cancer progression.

## Materials and methods

### Ethics statement

Written informed consent was obtained from all patients and their families before the study. Study protocols were approved by the Ethic Committee of Xiangyang Central Hospital, Affiliated Hospital of Hubei University of Arts and Science and based on the ethical principles for medical research involving human subjects of the Helsinki Declaration.

### Study subjects

A total of 75 melanoma tissues and their corresponding adjacent noncancerous tissues (≥ 5 cm surgical margins) as normal controls were collected from melanoma patients (between 2013 and 2018) in Xiangyang Central Hospital, Affiliated Hospital of Hubei University of Arts and Science. None of the patients received anti-cancer therapy before the surgical resection. Histological diagnosis of melanoma was evaluated according to criteria established by the World Health Organization (WHO) [19]. Part of the tissue samples was preserved in liquid nitrogen and part of them were fixed with 10% formalin. After routine dehydration, the tissues were preserved by paraffin embedding. The patients were followed up for 60 months and the survival was analyzed by the Kaplan–Meier method. During the follow-up period, the patient’s death was generally taken as the endpoint, otherwise, the final follow-up time would be the endpoint. The time interval from operation to death date was defined as the overall survival (OS).

### Cell treatment

Normal human skin melanocyte cell line PIG1, 4 malignant melanoma cell lines (A375, SK-MEL-1, SK-MEL-2, and OCM-1) and HEK-293T cells were purchased from the cell bank of the Chinese Academy of Sciences (Shanghai, China; http://www.cellbank.org.cn). The cells were cultured with Dulbecco’s modified Eagle’s medium (DMEM; 12800017, Gibco, Carlsbad, CA, USA) containing 10% fetal bovine serum (FBS; 26140079, Gibco, Carlsbad, CA, USA) and 1% penicillin/streptomycin in an incubator (BB15, Thermo Fisher Scientific Inc., Waltham, MA, USA) at 37 °C with 5% CO_2_. The culture medium was renewed every 24 h and passaged every 72 h. After removal of the medium, the cells were detached using 0.25% trypsin for 3 min, followed by the addition of the DMEM containing 10% FBS to terminate detachment and obtain a single cell suspension. After routine passage, the cells at the logarithmic growth phase were obtained and the expression of lncRNA GAS5 was assayed by reverse transcription quantitative polymerase chain reaction (RT-qPCR). The cells with the lowest expression of lncRNA GAS5 were applied for subsequent experiments. Cells were seeded into six-well plates for 24 h before transduction. When the cell confluence reached about 70%, the cells were transduced with the overexpression vector (oe) and shRNA plasmids of GAS5, EZH2, and CDKN1C or E2F4 shRNA plasmid, or treated with dimethyl sulfoxide (DMSO) (solvent for histone methylation inhibitors) or UNC1999 (specific inhibitor of histone methylase EZH2) using Lipofectamine 2000 (11668019, Thermo Fisher Scientific Inc., Waltham, MA, USA). In short, the cells were incubated with the mixture of transduction plasmids and 20 μL Lipofectamine 2000 diluted into 500 μL of serum-free medium for 48 h by adding with antibiotic-free DMEM containing 10% FBS. Subsequently, the interference efficiency of short hairpin RNA (shRNA) was assayed by RT-qPCR (sh-GAS5#1, sh-GAS5#2, sh-GAS5#3, sh-EZH2#1, sh-EZH2#2, sh-EZH2#3). The shRNA with the lowest expression of lncRNA GAS5 and EZH2 was selected for subsequent experiments. The full-length complementary deoxyribonucleic acid (cDNA) sequences of lncRNA GAS5, EZH2, CDKN1C (from the Ensembl database), and their respective negative control (NC) sequences (oe-GAS5 (G)-NC, sh-G-NC, oe-EZH2 (E)-NC, sh-E-NC, oe-CDKN1C (C)-NC, sh-C-NC) were designed using the lentiviral vector design software (Ambion, Company, Austin, TX, USA). All plasmids, vector construction, sequencing identification, virus packaging, and titer assay were performed by GENE Biotechnology Co., Ltd. (Shanghai, China).

### RNA isolation and quantification

Total RNA was isolated from tissues or cell samples using pre-cooled Trizol at 4 °C (Invitrogen, Carlsbad, CA, USA, USA) and reversely transcribed into cDNA using cDNA reverse transcription kit (K1622, Yaanda Biotechnology Co., Ltd., Beijing, China). Real-time fluorescence quantitative PCR was performed on a 7500-type quantitative fluorescence PCR (ABI Company, Oyster Bay, NY, USA) following the instructions of the SYBR^®^ Premix Ex Taq™ II kit (Takara, Tokyo, Japan). The glyceraldehyde-3-phosphate dehydrogenase (GAPDH) was employed as the internal reference while the expression of genes of interest was calculated using the 2^−ΔΔCt^ method (Table 1).

**Table 1:** Primer sequences for RT-qPCR

Gene	Forward sequence	Reverse sequence
LncRNA GAS5	5′-TCTGAGCAGGAATGGCAGTGT-3′	5′-CATCCTCCTTTGCCACAGAAC-3′
EZH2	5′-TCGAGCTGCTCTGCTCTCTA-3′	5′-CTTGAGCTGTCTCAGTCGCA-3′
CDKN1C	5′-CGTTCTTCTCGGGTGGA-3′	5′-CTGTACTCACTTGGCTCA-3′
GAPDH	5′-TGTTCGTCATGGGTGTGAAC-3′	5′-ATGGCATGGACTGTGGTCAT

RT-qPCR, reverse transcription quantitative polymerase chain reaction; LncRNA GAS5, long non-coding RNA growth arrest-specific transcript 5; EZH2, enhancer of zeste homolog 2; CDKN1C, cyclin-dependent kinase inhibitor 1C; GAPDH, glyceraldehyde-3-phosphate dehydrogenase; F, forward; R, reverse

### Western blot analysis

Total proteins were isolated from cells or tissues using the precooled radio-immunoprecipitation assay (RIPA) cell lysis buffer containing phenylmethylsulfonylfluoride (PMSF; R0010, Beijing Solarbio Science & Technology Co., Ltd., Beijing, China) at 4 °C. The bicinchoninic acid (BCA) kit (20201ES76, Yeasen Biotechnology Co., Ltd., Shanghai, Chins) was adopted to determine the protein concentration of each sample. The proteins were separated by polyacrylamide gel electrophoresis (PAGE), transferred onto the polyvinylidene fluoride (PVDF) membrane (Millipore Corporation, Bedford, MA, USA), which was sealed with 5% bovine serum albumin (BSA) at room temperature for 1 h. Subsequently, the membranes were incubated with the primary antibodies, rabbit polyclonal antibodies to EZH2 (ab195409, 1: 1000), inositol-requiring enzyme 1 (IRE1α; ab37073, 1: 2000), and superoxide dismutase-1 (SOD-1; ab13498, 1: 1000), rabbit monoclonal antibodies to E2F4 (ab150360, 1: 2000), histone 3 lysine 27 trimethylation (H3K27me3; ab192985, 1: 1000), CDKN1C (ab75974, 1: 500), and melanoma differentiation-associated genes 5 (MDA5; ab126630, 1: 3000), and GAPDH (ab181602, 1: 10000) at 4 °C overnight. After that, the membrane was re-probed with horseradish peroxidase (HRP)-conjugated secondary goat anti-rabbit antibody specific for immunoglobulin G (IgG; ab6721, 1:5000) for 1 h at room temperature, followed by the reaction of ECL resolution. The above described antibodies were all purchased from Abcam Inc. (Cambridge, UK). Finally, Quantity One v4.6.2 software was used for analysis with GAPDH as the internal reference.

### Dual-luciferase reporter gene assay

The target site sequence wild type (WT) in the 3′-untranslated region (3′-UTR) of CDKN1C mRNA and the sequence of the site-directed mutagenesis on the WT target site, mutant (MUT), were synthesized. Restriction endonuclease was employed for digestion of pmi-RB-REPORT™ plasmid (Guangzhou RiboBio Co., Ltd., Guangzhou, Guangdong, China). The synthesized target gene fragments WT and MUT were inserted into pmi-RB-REPORT™ vector (Guangzhou RiboBio Co., Ltd., Guangzhou, Guangdong, China). At the same time, the empty plasmid was transfected as the NC group. Vectors containing MUT and WT were co-transfected with overexpression (oe)-EZH2-NC or oe-EZH2 into HEK-293T cells, respectively. After 48 h of transfection, the relative light unit (RLU) was measured by a Renilla luciferase assay kit (YDJ2714, Shanghai Yuduo Biotechnology Co., Ltd., Shanghai, China). With firefly luciferase as the internal reference, the dual-luciferase reporter gene assay system (Promega Co, Madison, WI, USA) was employed for analysis.

### Fluorescence in situ hybridization (FISH) assay

The cells were seeded on a 24-well plate at the density of 6 × 10^4^ cells/well. When the cell confluence reached up to 60–70%, the cells were fixed by 4% paraformaldehyde containing 0.5% Triton X-100 for 10 min at room temperature followed by the blocking with 20 μL prehybridization solution (BREA-106, Beijing Biocreative Technology Co., Ltd., Beijing, China) per well at 37 °C for 30 min. Afterward, the cells were hybridized with Stellaris RNA FISH (Biosearch Technologies, Petaluma, CA, USA) probe hybridization solution targeting lncRNA GAS5 probes (lncRNA Gas5-1: gactcctacctcgaaaagac; lncRNA Gas5-2: agcaccatacctcacaggag) (NC sequence is scramble sequence) overnight at 37 °C under conditions void of light. The cells were then washed with washing solutions I, II, and III respectively at 42 °C. Subsequently, the cells were stained with 4′-6-diamidino-2-phenylindole (DAPI) solution for 10 min. Finally, the cell slides were sealed using anti-fluorescence quenching tablets (BIH0252, BioRike, Changsha, Hunan, China) and observed under a fluorescence microscope (Olympus, Tokyo, Japan).

### RNA immunoprecipitation (RIP) assay

The RIP kit (Millipore Corporation, Bedford, MA, USA) was adopted to examine the binding of lncRNA GAS5 and E2F4. The cells were lysed with RIPA lysis buffer (P0013B, Beyotime Institute of Biotechnology, Shanghai, China) and centrifuged at 14,000 rpm for 10 min at 4 °C. A part of the cell extract was taken out as an input and the remaining part was co-precipitated by incubation with the antibody. Next, 50 μL magnetic beads were resuspended in 100 μL RIP wash buffer and incubated with 5 μg antibody. The magnetic bead-antibody complex was resuspended in 900 μL RIP wash buffer and incubated with 100 μL cell extracting solution at 4 °C overnight. The sample was placed on the magnetic base and the magnetic bead-protein complex was collected. The sample and input were detached with proteinase K to extract RNA for subsequent western blot assay. The antibodies were recruited for RIP including the primary rabbit polyclonal antibody to E2F4 (ab245449, 1: 50, Abcam Inc., Cambridge, UK) and the rabbit anti-human IgG (ab109489, 1: 100, Abcam Inc., Cambridge, UK) as an NC.

### Chromatin immunoprecipitation (ChIP) assay

The enrichment of EZH2 and H3K27me3 in the promoter region of CDKN1C or E2F4 in the promoter region of EZH2 was observed using a ChIP kit (Millipore Corporation, Bedford, MA, USA). The A375 cells in the logarithmic growth phase were obtained. After obtaining about 70–80% of cell confluence, the cells were fixed with 1% formaldehyde at room temperature for 10 min to cross-link the DNA and protein. Afterward, the ultrasonic breaker was set to 10 s per ultrasonic cycle with 10^−s^ intervals with 15 cycles to break the chromatin and the cells were centrifuged at 13,000 rpm at 4 °C (a portion of DNA fragment as INPUT). The supernatants were collected and divided into three tubes, incubated overnight at 4 °C with the NC mouse antibody to IgG and target protein specific antibody, rabbit polyclonal antibody to EZH2 (ab195409, Abcam Inc., Cambridge, UK), rabbit monoclonal antibody to E2F4 (#40291, Cell Signaling Technologies, Beverly, MA, USA), rabbit monoclonal antibody to H3K27me3 (ab192985, Abcam Inc., Cambridge, UK). The endogenous DNA–protein complex was precipitated by Protein Agarose/Sepharose. After centrifugation, the supernatant was discarded and the non-specific complex was washed. Subsequently, the crosslinking was reversed overnight at 65 °C. The DNA fragment was extracted, purified, and recovered using phenol/chloroform with the INPUT as the internal reference. The CDKN1C gene promoter-specific primers are shown in Table 2.

**Table 2:** CDKN1C promoter-specific primers and 3′UTR non-specific primers

	Forward	Reverse
CDKN1C promoter	5′-GGTTGGGYGTTTTATAGGTTA-3′	5′-ACCTAACTATCCGATAATAAACTCTTC-3′
CDKN1C 3′-UTR	5′-ACAAGCACAAACAGACTGGAG-3′	5′-GACCGCCCCTCTCCTCGCAG-3′

CDKN1C, cyclin-dependent kinase inhibitor 1C

### RNA pull-down assay

A375 cells were transfected with 50 nM biotinylated WT-bio-GAS5 and MUT-bio-GAS5. After 48 h of transfection, the cells were incubated in specific lysis buffer (Ambion, Company, Austin, TX, USA) for 10 min and 50 mL sample cell lysate was then sub-packed. The remaining lysate was incubated with M-280 streptavidin beads (Sigma-Aldrich Chemical Company, St Louis, MO, USA) pre-coated with RNase-free and yeast tRNA (Sigma-Aldrich Chemical Company, St Louis, MO, USA) for 3 h at 4 °C. After being washed two times with pre-cooled lysis buffer, three times with low salt buffer, and one time with high salt buffer, the total protein was extracted using RIPA lysis buffer and E2F4 expression was assayed by western blot analysis.

### Enzyme-linked immunosorbent assay (ELISA)

The cells in the logarithmic growth phase were repeatedly frozen and thawed. After centrifugation at 2000 r/min for 15 min, the supernatant was collected to determine the reactive oxygen species (ROS) content. The standard sample was diluted and 15 standard wells and 3 blank wells were set on the enzyme labeled coated plate. Afterward, 40 μL sample diluent was added to blank wells and determination wells, and 10 μL sample to wells to be measured. After being sealed, the samples were incubated at 37 °C for 30 min, followed by incubation with 50 μL enzyme-labeled reagent (except blank well) at 37 °C for 30 min. After that, the cells in each well were developed with 50 μL chromogen A and 50 μL chromogen B at 37 °C for 15 min under conditions void of light. Finally, a 50 μL termination solution was added to terminate the reaction and the optical density (OD) values were measured at 450 nm. The standard curve was plotted and the linear regression equation was calculated with the standard concentrations as the X-axis and the OD values as the Y-axis. The concentration of ROS in samples was determined by the linear regression equation and then the actual concentration of ROS in samples was calculated as the method: [C (IU/mL) × dilution multiples ÷ sample protein concentration to be tested (mg/mL)].

### Cell counting kit-8 (CCK-8) assay

CCK-8 kit (CA1210-100, Beijing Solarbio Science & Technology Co., Ltd. (Beijing, China) was employed to assay the cell viability. The cells in the logarithmic growth phase were seeded into 96-well plates at a density of 5 × 10^3^ cells/well and incubated for 0, 24, 48, 72 and 96 h. The 10 μL CCK-8 solution was added, and the cells were incubated for 2 h before the OD values at 450 nm were determined by a microplate reader (BIO-RAD 680, Bio-Rad Laboratories, Hercules, CA, USA). The cell viability curves were plotted accordingly.

### Flow cytometric analysis

After 48 h of infection, the cells were detached with ethylene diamine tetraacetic acid-free trypsin and collected in the flow tubes and centrifuged. According to the Annexin-V-fluorescein isothiocyanate (FITC) apoptosis assay kit (Sigma-Aldrich Chemical Company, St Louis, MO, USA), the Annexin-V-FITC, propidium iodide (PI), and 4-(2-hydroxyethyl)-1-piperazineëthanesulfonic acid (HEPES) buffer solution was formulated into Annexin-V-FITC/PI staining solution at a ratio of 1: 2: 50. About 1 × 10^6^ cells were resuspended in 100 µL staining solution at room temperature for 15 min followed by the addition of 1 mL HEPES buffer. The 525 nm and 620 nm band-pass filters were excited at 488 nm to examine FITC and PI fluorescence respectively, followed by the observation of cell apoptosis.

### Statistical analysis

All data were analyzed using a Statistic Package for Social Science (SPSS) 21.0 statistical software (IBM-SPSS Inc., Chicago, IL, USA). The measurement data were described as mean ± standard deviation of three independent studies. The normality and homogeneity of variance were examined. The statistical significance of the unpaired data with normal distribution and equal variance was calculated using unpaired *t* test. Data among multiple groups were analyzed by one-way analysis of variance (ANOVA), followed by a Tukey’s post hoc test. The statistical analysis concerning time-based measurements within each group was realized using ANOVA of repeated measurements, followed by a Bonferroni’s post hoc test. Kaplan–Meier analysis was used for survival analysis and Pearson correlation analysis for correlation analysis. *p *< 0.05 was considered to be indicative of statistical significance.

## Results

### Poorly expressed lncRNA GAS5 in melanoma tissues and cells inhibits oxidative stress in melanoma

**Fig. 1:**
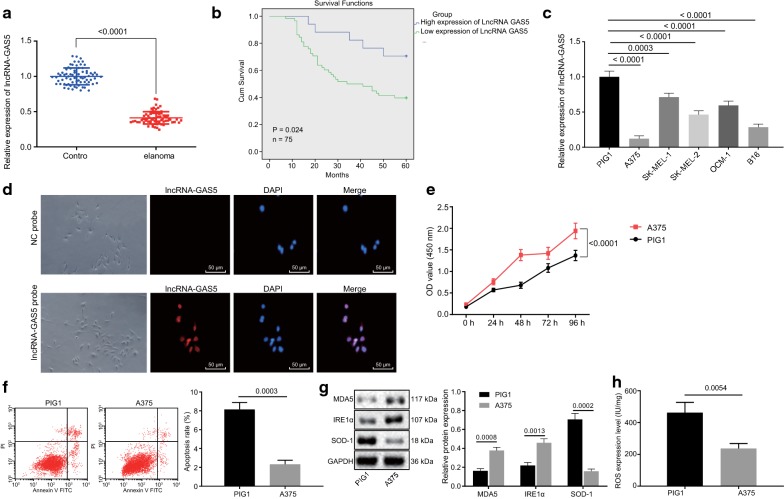
LncRNA GAS5 is poorly expressed in melanoma tissues and cells and inhibits oxidative stress in melanoma. A, RT-qPCR assay of lncRNA GAS5 expression in melanoma tissues and adjacent normal tissues. B, Survival time analysis by Kaplan–Meier method (n = 75). C, RT-qPCR screening of the cell line with the lowest expression of lncRNA GAS5. D, The FISH assay of subcellular localization of lncRNA GAS5 (200 ×). E, CCK-8 assay of A375 and PIG1 cell viability. F, Flow cytometric analysis of A375 and PIG1 cell apoptosis. G, Western blot analysis of protein expression of MDA5, IRE1α, and SOD-1 in A375 and PIG1 cells. H, ELISA analysis of ROS content in A375 and PIG1 cells. *p < 0.05, compared with the adjacent normal tissues or PIG1 cell. The above measurement data are expressed as mean ± standard deviation. The Paired *t*-test is adopted to analyze the data of melanoma tissues and adjacent normal tissues, and the unpaired *t*-test is used for comparison of the other data between two groups. Data among multiple groups are analyzed by one-way ANOVA, followed by Tukey’s post hoc test. The statistical analysis concerning time-based measurements within each group is realized using ANOVA of repeated measurements, followed by a Bonferroni’s post hoc test. Pearson correlation analysis is performed for correlation analysis. Kaplan–Meier analysis is used for survival analysis

To test whether the lncRNA GAS5 was dysregulated in melanoma cells, we performed RT-qPCR assays for lncRNA GAS5 in 75 melanoma tissues and adjacent normal tissues from patients with melanoma. It was found that lncRNA GAS5 exhibited lower expression in melanoma tissues than that in the adjacent normal tissues (*p *< 0.05; Fig. 1a), which helped to distinguish patients with low expression of lncRNA GAS5 and that with lncRNA GAS5 high expression. The correlation between lncRNA GAS5 expression and clinicopathological features in 75 cases of melanoma was analyzed, which suggested that the level of lncRNA GAS5 low expression in melanoma clinical specimens, was much higher than that of lncRNA GAS5 high expression (*p *< 0.05). LncRNA GAS5 expression was not correlated with gender and age (*p *> 0.05) but with the Breslow’s thickness of the tumor, ulceration, lymph node metastasis, and tumor-node-metastasis (TNM) staging (*p *< 0.05; Table 3). The survival analysis using the Kaplan–Meier method revealed that the OS of patients with low expression of lncRNA GAS5 was much lower than that with lncRNA GAS5 high expression (*p *< 0.05) (Fig. 1b). The expression of lncRNA GAS5 in the cultured 4 melanoma cell lines (A375, SK-MEL-1, SK-MEL-2, and OCM-1) and human normal skin melanocyte cell line (PIG1) was assayed using RT-qPCR. It was found that lncRNA GAS5 was expressed at a low level in A375, SK-MEL-1, SK-MEL-2, and OCM-1 cells compared with PIG1 and lowest in A375 cells. Therefore, A375 cells were selected for subsequent experiments (*p *< 0.05; Fig. 1c). The FISH assay was employed to further demonstrate that lncRNA GAS5 was primarily expressed in the nucleus (Fig. 1d), which was consistent with the predicted results of the lncATLAS website. These results indicated that lncRNA GAS5 was poorly expressed in melanoma tissues and cells were mainly localized in the nucleus. Additionally, the low expression of lncRNA GAS5 was associated with poor survival.

**Table 3:** Correlation analysis between the lncRNA GAS5 expression and the clinicopathological features of melanoma patients

Clinicopathological features	Cases (n = 75)	Expression of lncRNA GAS5		*p* value
		Low expression (n = 58, 77.33%)	High expression (n = 17, 22.67%)	
Gender				
Male	28	21 (75)	7 (25)	0.710
Female	47	37 (78.72)	10 (21.28)	
Age				
≤ 65 years	49	37 (75.51)	12 (24.49)	0.373
> 65 years	26	21 (80.77)	5 (19.23)	
Breslow thickness				
≤ 4 mm	18	6 (33.33)	12 (66.67)	0.001
> 4 mm	57	52 (91.23)	5 (8.77)	
Ulceration				
No	22	7 (31.92)	15 (68.18)	0.001
Yes	53	51 (96.23)	2 (3.77)	
Lymph node metastasis				
Negative	24	12 (42.86)	16 (57.14)	0.001
Positive	51	45 (97.83)	1 (2.17)	
TNM staging				
I	19	4 (21.05)	15 (78.95)	0.0001
II/III	56	54 (96.43)	2 (3.57)	

Data were measurement data, expressed by mean ± standard deviation. Data comparison was analyzed by Chi square test. *p* < 0.05 indicates significant difference

To further investigate the effect of lncRNA GAS5 expression on the biological processes of melanoma cells, A375, and PIG1 cells were selected as study subjects and western blot analysis was performed to examine the protein expression of MDA5, IRE1α, and SOD-1. The results from the CCK-8 assay further confirmed that A375 cell proliferation was accelerated (*p *< 0.05; Fig. 1e). Furthermore, flow cytometry revealed a decline in A375 cell apoptosis (*p *< 0.05; Fig. 1f). Interestingly it was observed that A375 cells exhibited an increased protein expression of MDA5 and IRE1α and diminished the protein expression of SOD-1 (*p *< 0.05; Fig. 1g). The ELISA displayed that the content of ROS in A375 cells was diminished (*p *< 0.05) (Fig. 1h), indicating the attenuation of oxidative stress. These above reported results displayed that the A375 cells with low expression of lncRNA GAS5 exhibited accelerated cell viability as well as suppressed oxidative stress and cell apoptosis.

### EZH2 overexpression accelerates oxidative stress in melanoma cells by targeting CDKN1C

**Fig. 2:**
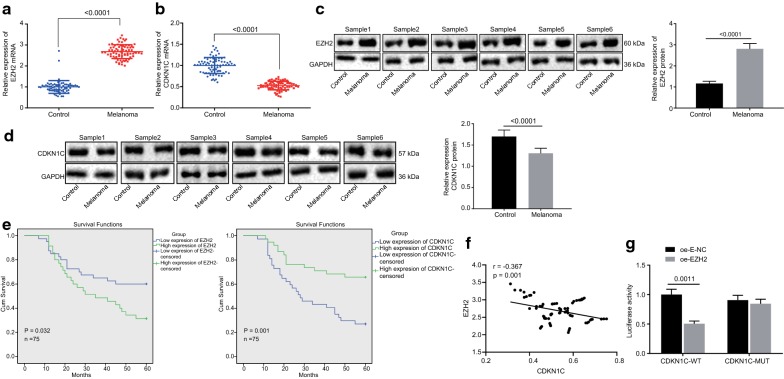
EZH2 overexpression accelerates oxidative stress in melanoma cells by targeting CDKN1C. **a**, **b**, RT-qPCR assay of mRNA expression of EZH2 (**a**) and CDKN1C (**b**) in melanoma tissues and adjacent normal tissues. **c**, **d**, Western blot assay of protein expression of EZH (**c**) and CDKN1C (**d**) in melanoma tissues and adjacent normal tissues. **e** Survival time analysis by Kaplan–Meier method (n = 75). **f** Correlation analysis of CDKN1C expression and EZH2 expression. G, Dual-luciferase reporter gene assay of the relationship between EZH2 and CDKN1C. *p < 0.05, compared with the adjacent normal tissues or cells transduced with oe-E-NC. The above measurement data are expressed as mean ± standard deviation. The Paired *t*-test is adopted to analyze the data of melanoma tissues and adjacent normal tissues, and the unpaired *t*-test is used for other data comparisons between two groups. Data among multiple groups are analyzed by one-way ANOVA, followed by Tukey’s post hoc test

Following after, RT-qPCR and western blot analysis were employed to examine the expression of EZH2 and CDKN1C in 6 pairs of melanoma tissues and adjacent normal tissues. It was found that EZH2 presented significantly higher expression in melanoma tissues than in adjacent normal tissues (Fig. 2a, c), while the expression of CDKN1C in melanoma tissues was lower than that in adjacent normal tissues (*p *< 0.05; Fig. 2b, d). Survival rate analysis carried out by the Kaplan–Meier method displayed that OS of patients with high expression of EZH2 or low expression of CDKN1C was much lower than OS of patients with low expression of EZH2 or high expression of CDKN1C (*p *< 0.05; Fig. 2e). Pearson correlation analysis (Fig. 2f) indicated that CDKN1C expression was reversely correlated with EZH2 expression (*p *< 0.001) suggesting, EZH2 could significantly inhibit the CDKN1C expression. The dual-luciferase reporter gene assay displayed that EZH2 could negatively regulate the transcriptional activity of the CDKN1C promoter region (*p *< 0.05; Fig. 2g) indicating that CDKN1C was a target gene of EZH2, which was consistent with Pearson correlation analysis. It could be concluded that EZH2 was highly expressed in melanoma cells while CDKN1C was poorly expressed. High expression of EZH2 or low expression of CDKN1C was associated with poor survival and CDKN1C was a target gene of EZH2.

**Fig. 3:**
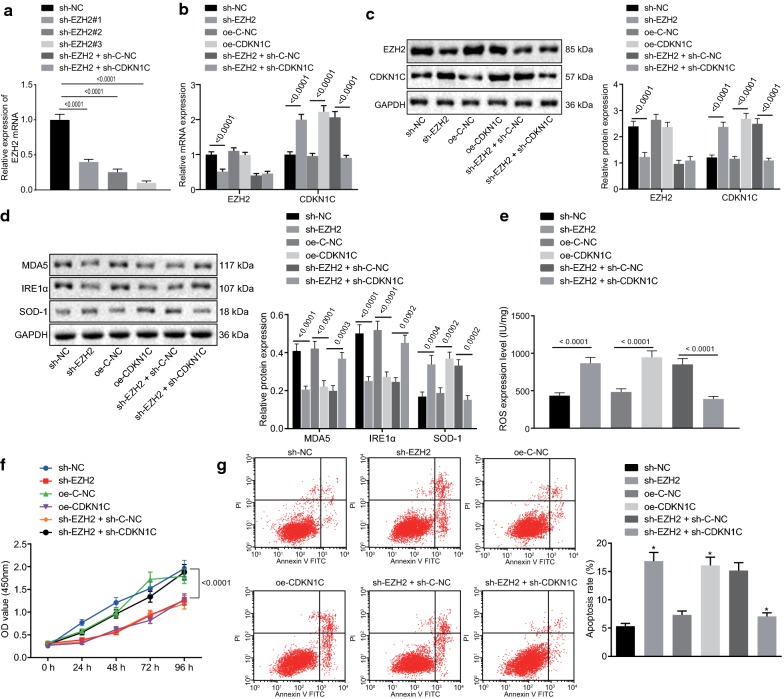
EZH2 down-regulates CDKN1C expression to inhibit oxidative stress in melanoma. **a** RT-qPCR assay of EZH2 silencing interference efficiency. A375 cells were treated with oe-CDKN1C, sh-EZH2 alone or in the presence of sh-CDKN1C. **b** RT-qPCR assay of mRNA expression of EZH2 and CDKN1C. **c** Western blot assay of protein expression of EZH2 and CDKN1C. **d** Western blot assay of protein expression of MDA5, IRE1α and SOD-1. **e** ELISA analysis of the content of ROS in transduced cells. **f** CCK-8 assay of cell viability. **g** Flow cytometric analysis of cell apoptosis. *p < 0.05, compared with the adjacent normal tissues, cells transduced with sh-E-NC, cells transduced with oe-C-NC or cells transduced with sh-EZH2 + sh-C-NC. #, the lowest silencing site of EZH2 expression. The above measurement data are expressed as mean ± standard deviation. The Unpaired *t*-test is used for other data comparisons between the two groups. Data among multiple groups are analyzed by one-way ANOVA, followed by Tukey’s post hoc test. The statistical analysis concerning time-based measurements within each group is realized using ANOVA of repeated measurements, followed by a Bonferroni’s post hoc test. Pearson correlation analysis is performed for correlation analysis

To investigate the effect of EZH2 and CDKN1C dysregulation on the biological process of melanoma cells, A375 cells were transduced with sh-E-NC, sh-EZH2, oe-C-NC, and oe-CDKN1C plasmids. RT-qPCR detection of the silencing efficiency displayed that the expression of EZH2 in the sh-EZH2#3 sequence group was the lowest (*p *< 0.05; Fig. 3a) thus, sh-EZH2#3 was selected for subsequent experiments. RT-qPCR and western blot analysis displayed that sh-EZH2 was successfully silenced EZH2 in A375 cells while oe-CDKN1C successfully overexpressed CDKN1C in A375 cells (*p *< 0.05) however, CDKN1C did not affect the expression of EZH2 (*p *> 0.05; Fig. 3b, c). Subsequently, western blot analysis was adopted to examine the protein expression of MDA5, IRE1α, and SOD-1. It was revealed that the silencing of EZH2 or overexpression of CDKN1C attenuated the protein expression of MDA5 and IRE1α while accelerated SOD-1 protein expression (*p *< 0.05; Fig. 3d). ELISA results suggested that the silenced EZH2 or over-expressing CDKN1C led to an increase of ROS content (*p *< 0.05; Fig. 3e), indicating that oxidative stress was activated. It was shown from the CCK-8 assay that downregulation of EZH2 or upregulation of CDKN1C suppressed the viability of A375 cells (*p *< 0.05; Fig. 3f). Flow cytometry unraveled that the silencing of EZH2 or overexpression of CDKN1C triggered the apoptosis of A375 cells (*p *< 0.05; Fig. 3g). The conclusion could be reached that silenced EZH2 or overexpressed CDKN1C could block the cell viability of melanoma and accelerate oxidative stress and cell apoptosis of melanoma.

A375 cells with silenced EZH2 expression were further transduced with sh-C-NC and sh-CDKN1C plasmids. RT-qPCR and western blot analysis displayed that sh-CDKN1C attenuated the expression of CDKN1C in A375 cells (*p *< 0.05) but CDKN1C did not affect the expression of EZH2 (*p *> 0.05; Fig. 3b, c). Western blot analysis of the protein expression of MDA5, IRE1α, and SOD-1 unraveled that the silencing of CDKN1C accelerated the protein expression of MDA5 and IRE1α while attenuated the SOD-1 expression (*p *< 0.05; Fig. 3d). ELISA results displayed that the silenced CDKN1C contributed to a decrease of ROS content (*p *< 0.05) (Fig. 3e), indicating that oxidative stress was blocked. It was shown from CCK-8 assay that the downregulation of CDKN1C accelerated the viability of A375 cells (*p *< 0.05; Fig. 3f). Flow cytometry displayed that the silencing of CDKN1C suppressed the apoptosis of A375 cells (*p *< 0.05; Fig. 3g). Therefore, it could be concluded that silenced CDKN1C accelerated the viability of melanoma cells and prevented melanoma cells from oxidative stress and apoptosis while EZH2 knockdown aided to improve these functions by upregulating the expression of CDKN1C.

### EZH2 overexpression diminishes CDKN1C expression by facilitating H3K27 trimethylation to attenuating oxidative stress in melanoma

**Fig. 4:**
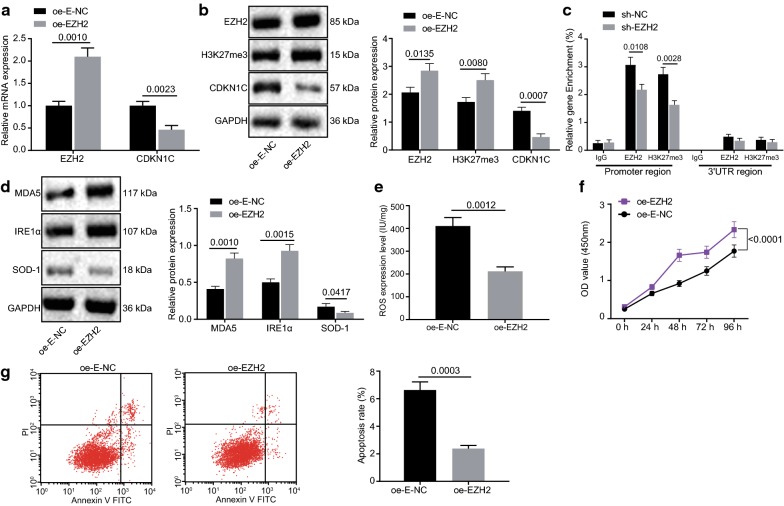
EZH2 overexpression diminishes CDKN1C expression by facilitating H3K27 trimethylation to attenuating oxidative stress in melanoma. **a** RT-qPCR assay of mRNA expression of EZH2 and CDKN1C. **b** Western blot assay of protein expression of EZH2, H3K27me3 and CDKN1C. **c** ChIP assay of EZH2 and H3K27me3 enrichment in CDKN1C promoter region. **d** Western blot assay of protein expression of MDA5, IRE1α and SOD-1. E, ELISA analysis of ROS content. F, CCK-8 assay of cell viability. G, Flow cytometric analysis of cell apoptosis. *p < 0.05, compared with the oe-E-NC group and the sh-E-NC group. The above measurement data are expressed as mean ± standard deviation. The unpaired *t*-test is used for comparison of the data between two groups. The statistical analysis concerning time-based measurements within each group is realized using ANOVA of repeated measurements, followed by a Bonferroni’s post hoc test

A375 cells were transduced with oe-E-NC and oe-EZH2 plasmids whereas the expression of EZH2, H3K27me3, and CDKN1C was examined by RT-qPCR and western blot analysis. It was found that oe-EZH2 was overexpressed in A375 cells (*p *< 0.05; Fig. 4a, b) and overexpression of EZH2 accelerated H3K27me3 levels while attenuated the CDKN1C expression (*p *< 0.05; Fig. 4a, b). The ChIP assay displayed that the level of H3K27me3 enrichment in the CDKN1C promoter region was notably diminished in cells with low expression of EZH2 (*p *< 0.05; Fig. 4c), indicating that CDKN1C was regulated by EZH2 and H3K27me3. Subsequently, western blot analysis was adopted to examine the protein expression of MDA5, IRE1α, and SOD-1. It was observed that overexpression of EZH2 accelerated the protein expression of MDA5 and IRE1α and attenuated SOD-1 expression (*p *< 0.05; Fig. 4d). Results from ELISA revealed that overexpression of EZH2 could lead to a decrease of ROS content (*p *< 0.05; Fig. 4e), indicating that oxidative stress was repressed. CCK-8 assay unraveled that overexpression of EZH2 accelerated the A375 cell viability (*p *< 0.05; Fig. 4f). Flow cytometry displayed that upregulating EZH2 prevented A375 cells from apoptosis (*p *< 0.05; Fig. 4g). Hence, it could be concluded that EZH2 lowers the expression of CDKN1C by facilitating H3K27 trimethylation thereby facilitating melanoma cell viability and inhibiting oxidative stress and apoptosis of melanoma cells.

### Overexpression of GAS5 reduces H3K27 trimethylation and upregulates CDKN1C expression to accelerate oxidative stress in melanoma by inhibiting EZH2

**Fig. 5:**
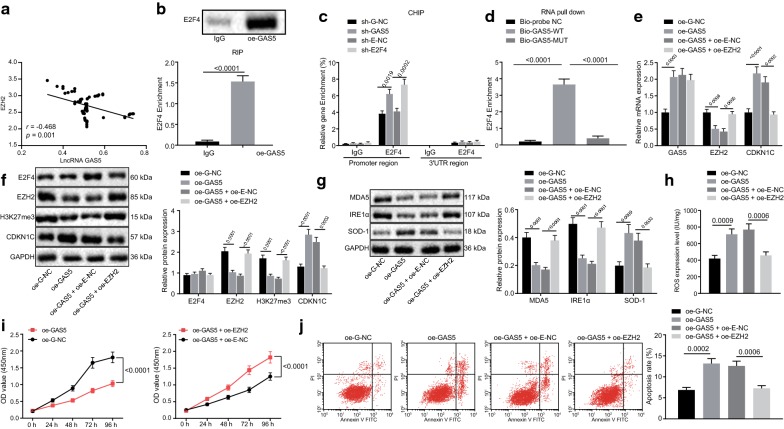
Overexpression of GAS5 prevents H3K27 trimethylation and upregulates CDKN1C expression to accelerate oxidative stress in melanoma by inhibiting EZH2. **a** Correlation analysis of lncRNA GAS5 expression and EZH2 expression. **b** RIP assay of the recruitment of transcription factor E2F4 by lncRNA GAS5. **c** ChIP assay of the enrichment of E2F4 in EZH2 promoter region. **d** RNA pull-down assay of the direct interaction of lncRNA GAS5 and E2F4. A375 cells were treated with oe-GAS5 alone or in the presence of oe-EZH2. **e** RT-qPCR assay of lncRNA GAS5 expression and mRNA expression of EZH2 and CDKN1C. **f** Western blot assay of the protein expression of E2F4, EZH2, H3K27me3 and CDKN1C. **g** Western blot assay of the protein expression of MDA5, IRE1α and SOD-1. **h** ELISA analysis of the content of ROS. **i** CCK-8 assay of cell viability. **j** Flow cytometric analysis of cell apoptosis. *p < 0.05, compared with the IgG group, cells transduced with oe-NC, Bio-probe NC group, cells transduced with oe-G-NC or cells transduced with oe-GAS5 + oe-E-NC. The above measurement data are expressed as mean ± standard deviation. The unpaired *t*-test is used for comparison of the data between two groups. Data among multiple groups are analyzed by one-way ANOVA, followed by Tukey’s post hoc test. The statistical analysis concerning time-based measurements within each group is realized using ANOVA of repeated measurements, followed by a Bonferroni’s post hoc test. Pearson correlation analysis is performed for correlation analysis

Pearson correlation analysis (Fig. 5a) displayed that lncRNA GAS5 expression was significantly negatively correlated with EZH2 expression (*p *< 0.001). RIP assay revealed E2F4 displayed a remarkable enrichment in cell lines with high expression of GAS5 (*p *< 0.05; Fig. 5b). Data obtained from the ChIP assay showed that E2F4 was markedly enriched in the E2F4 promoter region in cells with silenced lncRNA GAS5 and silenced E2F4 (*p *< 0.05; Fig. 5c), indicating that EZH2 was modulated by E2F4. Furthermore, RNA pull-down assay revealed that lncRNA GAS5 could directly interact with E2F4 (*p *< 0.05; Fig. 5d). Therefore, the conclusion could be reached that lncRNA GAS5 indirectly attenuated the EZH2 expression by the transcription factor E2F4 and affected the EZH2 expression from the transcriptional level.

Since overexpression of EZH2 accelerated H3K27 trimethylation leading to CDKN1C silencing and oxidative stress inhibition in melanoma. A375 cells were transduced with oe-G-NC and oe-GAS5 plasmids and melanoma cell-related biological changes were thus observed. The expression of lncRNA GAS5, E2F4, EZH2, H3K27me3, and CDKN1C was assayed using RT-qPCR and western blot analysis. It was found that GAS5 was overexpressed in A375 cells while overexpression of lncRNA GAS5 attenuated the expression of EZH2 and H3K27me3 and upregulated the expression of CDKN1C (*p *< 0.05; Fig. 5e, f) however, our results also showed that lncRNA GAS5 did not regulate the E2F4 expression (*p *> 0.05; Fig. 5e, f). Afterward, western blot analysis was adopted to examine the protein expression of MDA5, IRE1α, and SOD-1. It was found that overexpression of lncRNA GAS5 attenuated the protein expression of MDA5 and IRE1α, while accelerated the protein expression of SOD-1 (*p *< 0.05; Fig. 5g). ELISA revealed that overexpression of lncRNA GAS5 could lead to an increase of ROS (*p *< 0.05; Fig. 5h) suggesting the activation of oxidative stress. CCK-8 assay unraveled that overexpression of lncRNA GAS5 suppressed the A375 cell viability (*p *< 0.05; Fig. 5i). Flow cytometry revealed that the upregulation of lncRNA GAS5 accelerated the A375 cells apoptosis (*p *< 0.05; Fig. 5j). Therefore, it was indicated that overexpression of lncRNA GAS5 could block melanoma cell viability and stimulate the oxidative stress and apoptosis of melanoma cells.

A375 cells with overexpressing lncRNA GAS5 were further transduced with oe-E-NC or oe-EZH2 plasmids. RT-qPCR and western blot analyses were employed to examine the expression of lncRNA GAS5, E2F4, EZH2, H3K27me3, and CDKN1C and it was found that overexpression of lncRNA GAS5 suppressed the expression of EZH2 and H3K27me3 accelerated by oe-EZH2 and upregulated the CDKN1C expression (*p *< 0.05; Fig. 5e, f), yet lncRNA GAS5 did not mediate the E2F4 expression (*p *> 0.05; Fig. 5e, f). Western blot analysis was employed to examine the protein expression of MDA5, IRE1α, and SOD-1. It was found that overexpression of lncRNA GAS5, attenuated the upregulation of MDA5 and IRE1α protein expression induced by EZH2 and accelerated SOD-1 protein expression suppressed by EZH2 (*p *< 0.05; Fig. 5g). ELISA revealed that overexpression of lncRNA GAS5 resulted in a decline in ROS content caused by upregulation of EZH2 (*p *< 0.05) (Fig. 5h), suggesting the activation of oxidative stress. CCK-8 assay displayed that overexpression of lncRNA GAS5 attenuated the viability of A375 cells induced by the upregulation of EZH2 (*p *< 0.05; Fig. 5i). Flow cytometry revealed that upregulating lncRNA GAS5 accelerated a decrease in apoptosis of A375 cells induced by the upregulation of EZH2 (*p *< 0.05; Fig. 5j). Therefore, it could be concluded that overexpression of lncRNA GAS5 inhibits the EZH2 expression by recruiting E2F4 to EZH2 promoter, preventing H3K27 trimethylation, upregulating CDKN1C expression, thereby further inhibiting the melanoma cell viability and facilitating the oxidative stress and apoptosis of melanoma cells.

### UNC1999 inhibits H3K27 trimethylation and upregulates CDKN1C expression to accelerate oxidative stress in melanoma

**Fig. 6:**
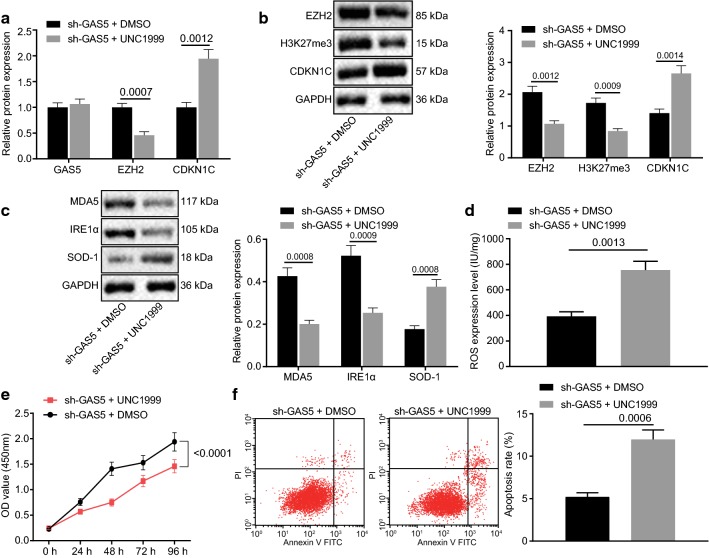
UNC1999 inhibits H3K27 trimethylation and upregulates CDKN1C expression to accelerate oxidative stress in melanoma. A375 cells were treated with sh-GAS5 in the presence of UNC1999 or DMSO. **a** RT-qPCR assay of lncRNA GAS5 expression and mRNA expression of EZH2 and CDKN1C. **b** Western blot assay of the protein expression of EZH2, H3K27me3 and CDKN1C. **c** Western blot assay of the protein expression of MDA5, IRE1α and SOD-1. **d** ELISA analysis of the content of ROS. E, CCK-8 assay of cell viability. F, Flow cytometric analysis of cell apoptosis. *p < 0.05, compared with the cells transduced with sh-GAS5 + DMSO. The above measurement data are expressed as mean ± standard deviation. The unpaired *t*-test is used for comparison of the data between two groups. The statistical analysis concerning time-based measurements within each group is realized using ANOVA of repeated measurements, followed by a Bonferroni’s post hoc test

Silencing lncRNA GAS5 accelerated the EZH2 expression as well as H3K27 trimethylation to suppress the CDKN1C transcription and oxidative stress in melanoma. A375 cells with lncRNA GAS5 silencing were added with DMSO or UNC1999 and the biological changes associated with melanoma cells were investigated by inhibiting the EZH2 expression and H3K27 methylation. The expression of lncRNA GAS5, EZH2, H3K27me3, and CDKN1C was assessed by RT-qPCR and western blot analysis. It was found that UNC1999 attenuated the expression of EZH2 and H3K27me3 while upregulated the expression of CDKN1C (*p *< 0.05; Fig. 6a, b) however, UNC1999 did not modulate lncRNA GAS5 expression (*p *> 0.05; Fig. 6a, b). The protein expression of MDA5, IRE1α, and SOD-1 was analyzed by western blot analysis. UNC1999 was found to suppress the protein expression of MDA5 and IRE1α and stimulated the protein expression of SOD-1 (*p *< 0.05; Fig. 6c). Results from ELISA demonstrated that the ROS content in the UNC1999 group was increased (*p *< 0.05; Fig. 6d) suggesting the activation of oxidative stress. CCK-8 assay displayed that A375 cell viability in UNC1999 group was attenuated (*p *< 0.05; Fig. 6e). Flow cytometry also exhibited that the A375 cells apoptosis in the UNC1999 group was accelerated (*p *< 0.05; Fig. 6f). Therefore, the conclusion could be reached that UNC1999 attenuated the methylation of H3K27me3 by inhibiting the expression of EZH2, restored the expression of CDKN1C, and reversed the melanoma cell viability induced by downregulation of lncRNA GAS5 and blocked the oxidative stress and apoptosis of melanoma cells.

## Discussion

Advanced melanoma has been implicated to link with a disappointing prognosis, resistance to cytotoxic chemotherapy, and the absence of treatment approaches [20]. The current progress in the treatment of melanoma mainly remained focused on exploring the function of the immune system as well as the oncogenic driver mutations in melanoma [21]. Previous evidence has indicated the role of lncRNA GAS5 in the progression of melanoma, which could be the possible indicators for melanoma and could serve as a therapeutic target [22]. EZH2 [23], as well as CDKN1C [24], have also been recognized as promising biomarkers for treating melanoma. Thus, the present study assessed the functions of lncRNA GAS5, EZH2, and CDKN1C in the oxidative stress of melanoma cells. Collectively, our investigation displayed that the silencing of lncRNA GAS5 and CDKN1C or elevation of EZH2, accelerated the viability of melanoma cells while suppressed oxidative stress and apoptosis of melanoma cells.

Initially, lncRNA GAS5 was found to be expressed at a poor level in melanoma tissues and cell lines and its deficiency attenuated the oxidative stress and apoptosis of melanoma cells. Zhang et al. have suggested a possible mechanism for LncRNA GAS5 as a tumor suppressor, which may be attributed to its ability in suppressing the oncogenic miR-21 in breast cancer [25]. Moreover, lncRNA GAS5 was downregulated in triple-negative breast cancer (TNBC) and elevation of lncRNA GAS5 has been verified to inhibit the proliferation of TNBC cells and accelerate apoptosis [26]. In another study, it was reported that knockdown of lncRNA GAS5 was observed to inhibit apoptosis as well as oxidative stress of melanoma cells and contribute to tumor progression in melanoma patients [27]. Intriguingly, MDA5 has been reported to display RNA-dependent ATPase activity while ectopic expression of MDA5 in human melanoma cells, inhibits the growth and differentiation of human melanoma cells [28]. However, oxidative stress was reported to lower the expression of MDA5 [29]. IRE1α knockdown in β cells is positively correlated with promoted oxidative stress by elevating over 300 mRNA expression of over-encoding functions [30]. The decreased level in SOD-1 ‘oxidative defense gene’, also further proved the reduction in oxidative stress [31]. Hence, these findings may provide a clue that lncRNA GAS5 was poorly expressed in melanoma and could be a possible candidate as tumor-inhibitor to affect the oxidative stress and apoptosis in melanoma cells. Another important finding of our study reported that the overexpression of EZH2 further accelerated the H3K27 trimethylation which suppress the oxidative stress as well as apoptosis in melanoma cell and stimulated the viability of melanoma cell via inhibition of CDKN1C. Consistently, previous study has reported that the elevated expression of EZH2 is found to be implicated in facilitating cell proliferation and oncogenic ability [32]. It has also been documented that EZH2 could have the potential to enhance the proliferation abilities of renal cell carcinoma cell line ACHN [33]. Additionally, EZH2 has been found to have a crucial role in facilitating the tumorigenesis of colon cancer [34]. EZH2 overexpression has been evidenced to play a significant role in stimulating melanoma progression [35]. Importantly, several studies reveal a critical role of EZH2 in repressing the expression of CDKN1C via activation of H3K27me3 and stimulation of its expression [36, 37]. CDKN1C has been identified as a tumor suppressor due to its diminished expression and functions of blocking biological signs of progress of breast cancer [38]. CDKN1C inhibition by miR-25 is also known to induce glioma cell proliferation and invasion [39]. In consent with these findings, our results showed that inhibition of CDKN1C by EZH2 leads to a significant increase in melanoma cell viability and a decrease in oxidative stress and apoptosis due to catalyzing the H3K27 trimethylation.

In subsequent experiments, we further identified that lncRNA GAS5 could upregulate CDKN1C to accelerate oxidative stress and apoptosis of melanoma cells and inhibit its viability by blocking EZH2 and H3K27 trimethylation. Similarly, Luo et al. Have reported that GAS5 acts as a tumor suppressor in prostate cancer development and progression via interacting with E2F1 and elevating the binding of E2F1 to the P27^Kip1^ promoter [40]. Sun et al. has also confirmed that expression of lncRNA GAS5 is markedly downregulated in gastric cancer tissues and the elevation of lncRNA GAS5 expression could inhibit cell proliferation and induce apoptosis partly via modulation of E2F1 and P21 expression [12]. Most notably, upregulated lncRNA GAS5 has been demonstrated to inhibit the transcription of EZH2 via recruitment of E2F4 to EZH2 promoter and induced cell apoptosis of bladder cancer [14].

## Conclusion

Consequently, our findings demonstrate that the elevation of lncRNA GAS5 upregulates CDKN1C to suppress the viability of melanoma cells and induce cell apoptosis as well as oxidative stress by inhibiting EZH2 expression and H3K27 trimethylation. Hence, these findings suggest that lncRNA GAS5 acts as an inhibitor in melanoma pathogenesis and provides a potential therapeutic target for melanoma treatment. However, the research is still at the preclinical stage whilst the role and underlying mechanism of lncRNA GAS5 in melanoma are not comprehensively investigated. Thus here we may suggest, that in the future study, a larger cohort of clinical samples should be included in the research to further determine the underlying mechanism of lncRNA GAS5, EZH2, and CDKN1C in melanoma.

## Data Availability

The datasets generated/analyzed during the current study are available.

## Abbreviations

lncRNAs: Long non-coding RNAs
GAS5: Growth arrest-specific transcript 5
PRC2: Polycomb repressive complex 2
EZH2: Enhancer of zeste homolog 2
CDKN1C: clin-dependent kinase inhibitor 1C
WHO: World Health Organization
OS: Overall survival
cDNA: Complementary deoxyribonucleic acid
GAPDH: Glyceraldehyde-3-phosphate dehydrogenase
PAGE: Polyacrylamide gel electrophoresis
WT: Wild type
RLU: Relative light unit
DAPI: Diamidino-2-phenylindole
ChIP: Chromatin immunoprecipitation
ELISA: Enzyme-linked immunosorbent assay
ROS: Reactive oxygen species
HEPES: Hydroxyethyl)-1-piperazineëthanesulfonic acid
SPSS: Statistic Package for Social Science
